# Notes and News

**Published:** 1897-10-23

**Authors:** 


### Notes and News.

(Collected and Communicated.)

Dr. Sanarelli is about to visit Italy, where he will lecture on several occasions on his reported discovery of a cure for yellow fever.

It is reported that the plague has spread to the Jullundur district of the Punjaub, and that 23 deaths have occurred already. The infection is believed to have been spread by pilgrims.

The Queen has sent £50 for the relief of the sufferers at Maidstone. The disease at last shows very real signs of abatement, and only six new cases were recorded in the last return. The pleasure fair usually held at this time of year was abandoned, but the cattle fair was held, though poorly attended.

On the 18th inst. the Duke of Cambridge visited Bath in order to lay the foundation-stone of the Victoria Library, and to open the magnificent new buildings which now constitute a most complete bath establishment. Many Continental processes have been introduced, including those of Nauheim. The Duke was enthusiastically received, and made himself a most genial and appreciative guest.

Vegetarian diet is no new thing, and the apostles of vegetarianism have now quite a large following. But of the extent of the applicability of the principles of vegetarianism very few of the unenlightened have any idea. In order to be more truly consistent, leather coverings for the feet have to be discarded by the cult, and vegetarian boots and shoes are now placed on the market. We can only hope that they will be found suitable to our wet streets, because the wearers may not resort to "something hot" if a cold results from wet feet.

The members of the Guild of St. Luke celebrated the thirty-third anniversary of the society on Wednesday evening by attending the festival in St. Paul's Cathedral. The service was instituted last year. The attendance this year was much larger. Between two and three thousand members of the Colleges of Surgeons and Physicians and of the Society of Apothecaries occupied the space under the dome. The members of the medical profession were clad in the robes attaching to their various degrees. Oxford and Cambridge were well represented, and the President of the Royal College of Physicians of Scotland was present. The musical service was rendered by the London Gregorian Choral Association, with a choir numbering two hundred. The Archbishop of York preached the sermon on the text, "Himself took up our infirmities and bare them."

The foundation-stone of the Convalescent Home at Bognor for the East London Hospital for Children will be laid by Thomas Dixon Galpin, Esq., on Thursday, October 28th, at 1.30 p.m.

Alderman Strutt is presenting the Derby Royal Infirmary with a new convalescent home. The building is to accommodate 28 patients. The front portion is to be two storeys high, with a central water tower. The corridors and staircases are to be fireproof. Mr. Strutt gives £5,000 for building purposes, and £2,000 as endowment.

The plethora of great men in the world of medicine seems to be as marked in America as it is in England. Professors must, indeed, abound in that happy land, for we are told of one medical college in which there are 38 professors, 23 assistant professors and instructors, and 51 students, giving one professor or instructor to each student, with ten left over.

In pursuance of the known wishes of the late Mr. B. I. Barnato, the firm of Barnato Brothers have made the following donations to hospitals: To the London Hospital, £2,000, to endow two cots to be called after him; St. Mary's Hospital, £1,000, to endow one cot; the same to Charing Cross Hospital, St. George's Hospital, the Cancer Hospital, the Metropolitan Hospital, and the City Orthopædic Hospital. The Hospital and Home for Incurable Children is to have £250. The Jewish charities and many others benefit also in a distribution of £16,300 in all.

The amount of money contributed by the public for the relief of suffering in this Jubilee year is truly remarkable. The Indian Famine Fund alone secured upwards of £550,000, the Hospital Sunday Fund obtained £40,000, the Princess of Wales' Jubilee Fund £30,000, the Prince of Wales' Hospital Fund upwards of £150,000, the Maidstone Typhoid Relief Fund upwards of £12,000, and the Montserrat Relief Fund £1,500. These sums are all collections. The charity given privately by individuals is not included, and is also very large.

Sir Sydney H. Waterlow, Bart, Chairman of the distribution Committee of the Hospital Sunday Fund, will read a paper on "The System of Distribution of the £913,482 raised on Hospital Sunday in London, 1873-97," before the Hospitals Association, in the Board Room of Charing Cross Hospital, on Thursday, October 28th, at four p.m. Mr. Acland, Chairman of the Hospital Saturday Fund, will open the discussion and explain his method of distribution. The chair will be taken by Mr. H. Cosmo Bonsor, M.P., Treasurer of Guy's Hospital. Tickets may be obtained on application to the honorary secretary, at 28, Southampton Street, Strand, W.C.

It is a matter of much satisfaction at Penrith that a cottage hospital is to be erected. The occasion of the Jubilee was utilised to awaken public interest and generosity, with the result that £1,500 has been subscribed, and a good number of annual contributions promised. Lord Lonsdale has given the site, and the institution, which is to serve Penrith and the surrounding district, will contain two wards, nurses' room, bedrooms, and offices. The property is to be vested in trustees, a committee of management will be elected by subscribers, and payments are to be made by patients according to circumstances.

